# A Pre-Clinical Safety Evaluation of SBP (HBsAg-Binding Protein) Adjuvant for Hepatitis B Vaccine

**DOI:** 10.1371/journal.pone.0170313

**Published:** 2017-01-19

**Authors:** Jingbo Wang, Caixia Su, Rui Liu, Baoxiu Liu, Inam Ullah Khan, Jun Xie, Naishuo Zhu

**Affiliations:** Laboratory of Molecular Immunology, State Key Laboratory of Genetic Engineering, Institute of Biomedical Science, School of Life Sciences, Fudan University, Shanghai, China; Centre de Recherche en Cancerologie de Lyon, FRANCE

## Abstract

Although adjuvants are a common component of many vaccines, there are few adjuvants licensed for use in humans due to concerns about their toxic effects. There is a need to develop new and safe adjuvants, because some existing vaccines have low immunogenicity among certain patient groups. In this study, SBP, a hepatitis B surface antigen binding protein that was discovered through screening a human liver cDNA expression library, was introduced into hepatitis B vaccine. A good laboratory practice, non-clinical safety evaluation was performed to identify the side effects of both SBP and SBP-adjuvanted hepatitis B vaccine. The results indicate that SBP could enhance the HBsAg-specific immune response, thus increasing the protection provided by the hepatitis B vaccine. The safety data obtained here warrant further investigation of SBP as a vaccine adjuvant.

## Introduction

The infectious disease hepatitis B, which is caused by the hepatitis B virus (HBV), has troubled people worldwide for many years. The current prophylactic HBV vaccines, which are based on recombinant hepatitis B surface antigen (HBsAg), have successfully decreased rates of HBV infection and transmission. However, there are more than 2 billion people who have been infected with HBV and are therefore at high risk for liver failure, cirrhosis, or cancer [1]. Specific groups of patients respond poorly or not at all to conventional HBV vaccines. Because of the poor immunogenicity of HBsAg, new methods are needed to improve the ability of the HBV vaccine to trigger protective immunity [2, 3]. Third-generation HBV vaccines that combine small S antigen with PreS1 and PreS2 antigens have been shown to induce a stronger immune response in non- and low responders than current HBV vaccines [4]. Conventionally used adjuvants, such as aluminum salts, allow for persistent release of the antigen, delaying clearance and resulting in more exposure to the immune system [5]. Adjuvants can elicit effective innate and adaptive immune responses through increasing the ability of antigens to activate signaling pathways.

Although adjuvants improve vaccine formulations and lead to better and more controllable immune responses, very few adjuvants have been licensed for use in humans because of concerns about side effects. Approved adjuvants include aluminum hydroxide, the oil-in-water emulsions MF59 [6] and AS03 [7], and alum with monophosphoryl lipid A (AS04) [8]. Although alum is considered a safe adjuvant in humans, it has been associated with local reactions and increased IgE antibody responses [9]. Because of these limitations, there is an important need to identify novel adjuvants for HBV vaccines.

The novel HBsAg-binding protein, SBP, has been screened from a human liver cDNA expression library. Previous results show that, when combined with an HBV vaccine, SBP can promote the uptake of HBsAg by antigen-presenting cells and enhance HBsAg-specific antibody production in BALB/c mice without any noticeable side effects [10]. These results suggest that SBP has the potential to be used as a novel adjuvant for HBV vaccine. We have been developing a formulation of HBV vaccine consisting of HBsAg and SBP. The pharmacodynamics and safety of this new vaccine are evaluated normatively in the study presented here.

## Materials and Methods

### Study design and vaccines

**Fig 1:**
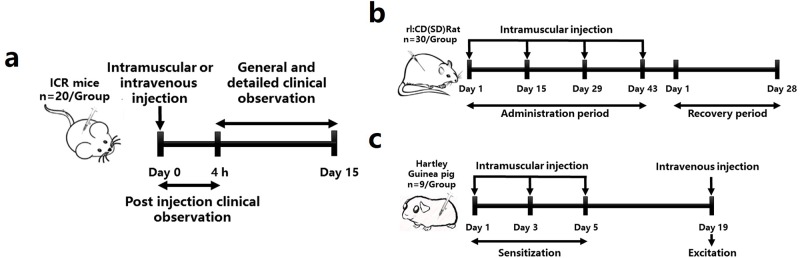
Procedures of safety evaluation. (a) Experimental design of GLP non-clinical acute toxic test in ICR mice. (b) Experimental design of GLP non-clinical long toxic test in rats. (c) Experimental design of GLP non-clinical allergic test in guinea pigs.

We undertook a GLP pre-clinical allergic reaction test, acute and long term toxicity test [11, 12] to evaluate the safety of SBP and a candidate hepatitis B vaccine (Fig 1). SBP, HBsAg and new hepatitis B vaccine were produced by Dalian Hissen Bio-pharmaceuticals Company (Dalian, China). Each dose of SBP adjuvanted vaccine (H-S, 0.5ml, Lot NO. 201501013S115) was a mixture of HBsAg (10μg), SBP (15μg) and aluminum (0.22–0.3 mg). The concentration of SBP solution (S, Lot NO. 20141212) was 30 μg/ml and each dose of general vaccine (H, 0.5ml, Lot NO. 201501013) was a mixture of HBsAg (10μg) and aluminum (0.22–0.3 mg).

### Animals and procedures

#### Animal procurement and care

Sprague Dawley (SD) rats for pharmacodynamics studies were purchased from the Shanghai SLAC Laboratory Animal Company (Shanghai, China) and maintained under specific pathogen free (SPF) conditions at Fudan University. For the good laboratory practice (GLP) safety evaluation, SD rats, Institute of Cancer Research (ICR) mice, and Hartley guinea pigs were purchased from Beijing Vital River Laboratory Animal Technology (Beijing, China) and maintained under SPF conditions at Beijing JOINN laboratories. *Macaca fascicularis* for GLP pharmacodynamics and safety studies were purchased from and maintained under SPF conditions at the National Shanghai Center for Drug Safety Evaluation and Research. All procedures were performed according to the guidelines established by the National Institutes of Health, and every effort was made to minimize discomfort and suffering on the part of the animals. The GLP study was approved by the China Food and Drug Administration, and the other animal studies were approved by the Animal Experiment Committee of Fudan University. All principles of laboratory animal care were followed precisely.

#### Pharmacodynamics study

For the pharmacodynamics study, 60 SD rats (7–8 weeks of age) were randomly divided into 6 groups. Each rat received intramuscular injections 3 times at 2-week intervals. For each rat, the injections contained one of the following dosage: 1.25 μg HBsAg, 2.5 μg HBsAg, 10 μg HBsAg, 1.25 μg HBsAg + SBP, 2.5 μg HBsAg + SBP, or 10 μg HBsAg + SBP. Serum samples were collected on days 0, 7, 14, 21, 28, 58, 88, and 118 following the first injection to observe the production of HBsAg-specific antibodies.

#### Acute toxicity study

Eighty ICR mice (4–5 weeks of age) were divided into 4 groups. Because the clinical administration of HB vaccine is intramuscularly injection, we choose intramuscularly injection as the major administration of drugs. Furthermore, we add intravenously injection of SBP group to evaluate the side effect of this new adjuvant. Each mouse received an injection of one of the following: normal saline, SBP (0.5 mL, intramuscularly injection), SBP (0.5 mL, intravenously injection) or HBsAg + SBP (0.5 mL, intramuscularly). To determine if any acute toxic reactions occurred, the animals were observed continuously for 4 h after the injection was administered. General and detailed clinical observation continued for 15 d thereafter. Then all animals were sacrificed and gross anatomy was performed to observe the lesion of different organs.

#### Long-term toxicity test

To study long-term toxic effects of the adjuvant, 150 SD rats (half males and half females, 7–8 weeks of age) were divided into 5 groups. Each rat was injected intramuscularly on days 0, 15, 29, and 43 with normal saline, SBP (0.5 mL or 1.5 mL), or HBsAg + SBP (0.5 mL or 1.5 mL). Various parameters, including clinical signs, body weight, and temperature, were monitored to evaluate the possibility of long-term toxic reaction to SBP and vaccine. Blood samples were collected on days 3, 46, and 72 for hematology and biochemistry assays. Three days after the last injection, 20 rats (half males and half females) from each group were sacrificed for gross and histopathological examination. The remainder of the rats were sacrificed at the end of the recovery period for gross and histopathological examination.

#### Allergic reaction test

For the allergic reaction test, 54 male Hartley guinea pigs were divided into 6 groups. Guinea pigs were injected intramuscularly with normal saline (0.5 mL), human albumin (0.5 mL, 40 mg/mL), SBP (0.05 mL or 0.5 mL), or HBsAg + SBP (0.05 or 0.5 mL) 3 times at 2-day intervals in order to sensitize them to the corresponding antigens. Fourteen days after the last injection, each guinea pig was injected intravenously with a double dosage of the corresponding antigens and observed continuously for 30 min to record allergy symptoms.

#### *Macaca fascicularis* study

To evaluate the effects of SBP in *Macaca fascicularis*, animals were injected on days 1, 9, 15, and 32 with HBsAg (0.5 mL) or HBsAg + SBP (0.5 mL). Blood samples were collected on days 0, 8, 14, 20, 31, and 37 for hematology, antibody titer, and lymphocyte differentiation analysis.

### Enzyme linked immunosorbent assay

The HBsAg specific serum antibody was measured using ELISA. Briefly, 100 μl/well diluted serum samples or rat antibody standards (Abcam, Cambridge, UK) were added into a 96-well plate, and following by incubation with HRP conjugated goat anti-mouse IgG antibodies (Santa Cruz, CA, USA). After washing, 100 μl/well TMB substrate (BD Biosciences, San Diego, CA, USA) was added and the reactions were terminated by the addition of 50 μl/well 2M H2SO4. The absorbance at 450nm was immediately read on a microplate reader (Molecular devices, Sunnyvale, CA, USA). A standard curve of IgG standard was used to transform the absorbance to antibody concentration.

### Flow cytometry

For lymphocytes differentiation, blood samples were harvested at indicated time points and monocytes were separated though isodensity centrifugation. Thereafter, approximately 10^6^ cells were re suspended in 100 μL of PBS and incubated with 2 μg anti-CD3c FITC together with anti-CD4 PE or anti-CD8 PE antibodies (all from BioLegend, San Diego, CA, USA) for 20 min on ice. Thereafter, the cells were washed twice with PBS and flow cytometry was performed using a FACSort instrument (BD Biosciences, San Jose, CA, USA). Data was analyzed using FlowJo software (Tree Star, San Caros, CA, USA).

### Statistical analysis

The statistical significance of the results was determined using the Provantis system (SAS, US). The homogeneity of variance was examined with Levene’s test. A one-way ANOVA was used to determine the significance of the data (*P* > 0.05). For groups in which *P* ≤ 0.05, Dunnett’s test was used for comparative analysis. For groups with heterogeneity of variance, Levene’s test was used after log transforming the data. The Kruskal-Wallis test was used for data that still had heterogeneity of variance after log transformation. When evaluating differences between experimental and control samples, *P* ≤ 0.05 was considered to be statistically significant.

## Results

### SBP adjuvant enhances HBsAg-specific antibody production in rats

**Fig 2:**
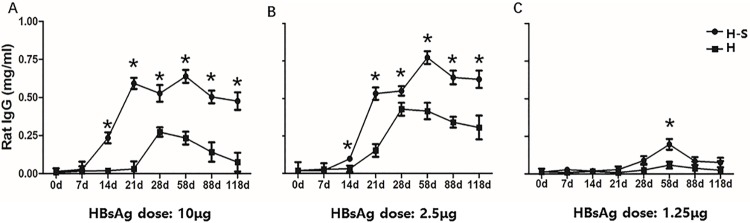
HBsAg specific antibody induced by SBP adjuvanted and non-adjuvanted vaccines. Rats (n = 10/group) were immunized intramuscularly 3 times at a 2-week interval with vaccine alone (H) or together with SBP (H-S). There are 6 groups with 3 injected HBsAg dose: 1.25μg, 2.5μg and 10μg. Blood samples were collected on indicated time points and anti-HBs total IgG was measured by ELISA. Results are expressed as the means ± SEM, * p<0.05.

The serum antibody titer correlates with the protection offered by vaccines. To determine the effects of SBP on the immunogenicity of HBV vaccine, HBsAg-specific antibody was assessed by enzyme-linked immunosorbent assay in rats that received various dosages of HBsAg with or without the SBP adjuvant. Higher dosages of HBsAg adjuvanted with SBP triggered more IgG generation in rats after day 14, and IgG production doubled on day 21 compared with the non-adjuvanted group (Fig 2). For the lowest dosage group, an increase in IgG was first detected on day 21, and the rats that received SBP had higher levels of IgG production. The results also show that SBP plays a role in the maintenance of antibody levels over time. In the HBsAg group, anti-HBV IgG began to decline in the second month, while the antibody levels of the groups that received vaccine adjuvanted with SBP did not decline until the third month.

### SBP and vaccine did not induce acute toxicity in ICR mice

Before clinical safety evaluation, vaccine development requires extensive pre-clinical testing in animals. Institute of Cancer Research mice were used to evaluate the acute toxicity effects of SBP and SBP-adjuvanted HBV vaccine. No mice died during the study, and no clinical abnormalities in mental state, behavior, respiration, morbidity, secretions, feces, skin, eyes, ear, stomach, etc. were observed in the treated mice. There were also no significant differences between groups in body weight or food/water consumption (data not shown). The gross anatomy of each mouse was examined after they were sacrificed, and no abnormalities were observed in the main organs including brain, skin, liver, spleen, and kidneys.

### SBP did not induce long-term toxicity in rats, but high dosages of SBP-adjuvanted vaccine could cause local irritation

**Fig 3:**
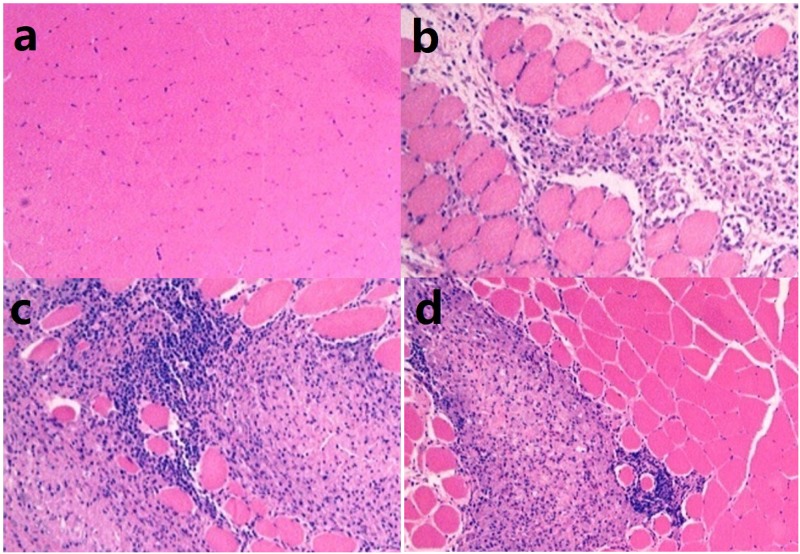
SBP could induce local irritation reaction. Representative photomicrographs of treated site are shown with original magnification: × 200 (HE staining). (a) NS group, 3 days after the last injection, normal tissue. (b) V+SBP (high dose) group, 3 days after the last injection, necrosis of myofiber (focal). (c) V+SBP (high dose) group, 3 days after the last injection, mesenchyme inflammatory cell infiltration and macrophage aggregation (multi-focus). (d) V+SBP (high dose) group, at the end of the recovery period, mesenchyme inflammatory cell infiltration and macrophage aggregation (multi-focus).

For the long-term toxicity study, SD rats were injected repeatedly with SBP alone or SBP in combination with HBV vaccine. Generally, each dosage of SBP did not result in abnormal changes in any of the tested parameters (Table 1). For the groups that received vaccine, common irritation reactions, such as necrosis of the myofiber, were observed at the injection site. As shown in Fig 3, lesions were still present at the end of the recovery period, indicating that the condition would likely take a long time to resolve.

**Table 1:** Preliminary GLP pre-clinical long term toxicity tests in rats.

Index	Administration period	Recovery period
**Mortality**	1 death in H-S group (overdose anesthesia)	None
**Clinical signs**	No abnormalities	No abnormalities
**Body weight**	No difference	No difference
**Food consumption**	Normal	Normal
**Temperature**	Slightly change at 3 time points	Slightly change at 1 time point
**Ophthalmic testing**	No abnormalities	No abnormalities
**Organ-body ratios**	No abnormalities	No abnormalities
**Gross anatomy**	White nodule at injection part in H-S group	White nodule at injection part in H-S group
**Injection part**	Necrosis of myofiber and mesenchyme inflammatory cell infiltration, macrophage aggregation and abscess in H-S group	Macrophage aggregation and mesenchyme inflammatory cell infiltration in H-S group

Hematology results from the long-term toxicity test showed that female rats in the SBP-adjuvanted vaccine (0.5 mL) group had increased levels of neutrophils (cells important in innate immunity and anti-inflammation) 3 days after the final injection. Male rats in the SBP-adjuvanted vaccine (1.5 mL) group showed decreased levels of neutrophils, eosinophils (a type of white blood cell that combats multicellular parasites and allergens), and monocytes (the largest type of leukocyte, which can phagocytize foreign materials and play a role in immune response) (*P* < 0.05) (Tables A-D in S1 File). The inflammatory changes at the injection sites (Fig 3) may be related the immunoreaction caused by the vaccine. Three days after the first injection, the level of reticulocytes (immature red blood cells which could reflect the red blood hematopoietic function of bone marrow) decreased in female rats for the SBP (0.5 mL) group, and the mean corpuscular hemoglobin concentration (often used to identify anemia) decreased in male rats from the SBP (1.5 mL) and SBP-adjuvanted vaccine (0.5 mL) groups (data not shown). Although these changes were statistically significant, there was no clear correlation with injection dosage or time. It is likely that these data do not have toxicological meaning.

### SBP and high-dosage SBP-adjuvanted vaccine could cause allergic reaction in guinea pigs

The allergenicity of SBP and SBP-adjuvanted vaccine was evaluated in guinea pigs. No abnormalities were observed during the sensitization period. After the antigens were administered, different levels of active systemic allergic reaction were observed in both SBP groups. Furthermore, the higher dosage of SBP-adjuvanted vaccine also caused active systemic allergic reactions (Table 2).

**Table 2:** GLP allergic reaction test in guinea pig.

Group	Positive rate	Extent of reaction
**Negative control**	0	-
**Positive control**	1	+~+++
**S (0.5ml)**	0.44	+~++
**S (1.5ml)**	1	+~++++
**H-S (0.5ml)**	0	-
**H-S (1.5ml)**	0.67	+~+++

–, negative allergy; +, weakly positive allergy; ++, positive allergy; +++, strong positive allergy; ++++, extremely positive allergy.

### SBP could induce the production of SBP-specific antibodies in rats

**Fig 4:**
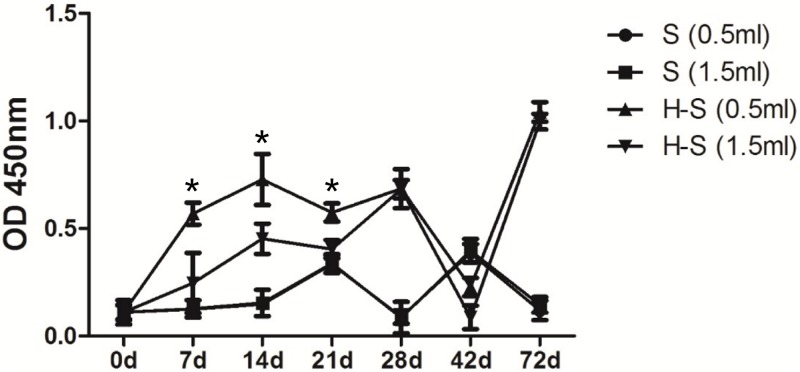
SBP-specific antibody production in rats. Rats (n = 10) were immunized intramuscularly 3 times at a 2-week interval with vaccine alone (H) or together with SBP (H-S). Blood samples were collected on indicated time points and anti-SBP total IgG was measured by ELISA. Results are expressed as the means ± SEM. * p<0.05.

As a foreign protein for rats, SBP could induce antibody production after injection. In both SBP groups, SBP-specific antibodies were detected on day 21, after 2 injections, and declined 7 days afterwards (Fig 4). For SBP-adjuvanted vaccine groups, the lower dosage appeared to induce higher antibody production according to our results. It seems that the SBP-specific antibody was produced, and then levels declined in a dosage-independent manner. The detailed mechanism is not yet clear.

### SBP-adjuvanted HBV vaccine could induce stronger immune response in *Macaca fascicularis* than unadjuvanted vaccine, without any side effects

**Fig 5:**
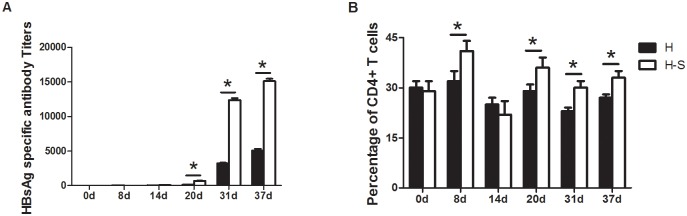
SBP adjuvanted hepatitis B vaccine could induce higher antibody titers and higher percentage of Th cells. Macaca fascicularis were immunized intramuscularly at days 1, 9, 15, 32 with vaccine alone (H) or together with SBP (H-S). (A) Blood samples were collected on indicated time points and HBsAg specific antibody titers were measured. (B)Percentage of CD4+ T cells was also measured at these time points. Results are expressed as the means ± SEM, * p<0.05.

*Macaca fascicularis* were used to determine the effects of SBP-adjuvanted vaccine in primates. As shown in Fig 5, animals that received SBP-adjuvanted vaccine had higher antibody levels after day 30 than the animals that received non-adjuvanted vaccine. This suggests that SBP could be a good adjuvant to the HPV vaccine.

T cell—dependent immune responses were also important to antibody production. Our results showed that the animals who received the SBP-adjuvanted vaccine had slightly elevated levels of CD4+ T cells, which suggests that SBP may promote the differentiation and maturation of T cells. However, the levels of T cells, and other hematological parameters, were within the normal range and the differences between the groups was not statistically significant (data not shown).

## Discussion

Although the existing HBV vaccine provides many people with significant protection against infection [13], a more effective vaccine is required for poor-response groups [14, 15]. In a previous study, we found an HBsAg-binding protein that could promote the immune process both in vitro and in vivo. Its adjuvanticity in connection with several antigens (HBsAg, rabies [16], and rotavirus [17]) has been evaluated in mice and the results indicate that it has the potential to be a new adjuvant for use in human vaccines.

During vaccine and adjuvant development, various pre-clinical testing is required before clinical safety evaluation [12, 18]. It is very important to evaluate potential side effects via toxicology tests in animals. Standard GLP non-clinical pharmacology, toxicity, and allergy studies for SBP and SBP-adjuvanted HBV vaccine were completed in rodents in the present study. The results demonstrate that SBP-adjuvanted HBV vaccine could enhance the production of anti-HBsAg antibody significantly. Throughout both acute (single dosage) and sub-acute (repeated dosage) studies, SBP and SBP-adjuvanted vaccine were well-tolerated. No treatment-related effects in food consumption, body weight, clinical signs, body temperature, ophthalmic testing, or organ-body weight ratios were noted.

White nodules were found at the injection site in the repeated-dosage vaccine group 3 days after the final injection. Histopathological examination of the nodules showed myofiber necrosis, mesenchyme inflammatory cell infiltration, macrophage aggregation, and abscesses. Major mechanical stimulation, including myofiber necrosis and mesenchyme inflammatory cell infiltration, was also observed in SBP group. Injection site reactions such as these are not uncommon with adjuvanted vaccines: Lawrence Segal et al. reported that human papillomavirus vaccine could induce injection site reactions in rabbits and rats [18], and Penina Haber et al. showed that inactivated influenza vaccine induced injection site reactions in pre-licensure clinical trials [19, 20]. Some statistically significant changes in blood biochemistry parameters were observed in the vaccine group 3 days after the final injection, which we considered to be associated with the inflammatory response at the injection site. All other differences were considered incidental and within the normal reference ranges for the animals, and therefore of no toxicological significance.

Allergic sensitization of drugs is an important health problem with serious clinical consequences [21, 22]. In the pre-clinical evaluation process, guinea pig models are often used to evaluate the allergenicity of drugs [23, 24]. Our results show that SBP could induce strong systemic active allergic reaction under the conditions used in this study. Considering that SBP has been shown to be an immunogenic protein for guinea pigs at a sensitization dosage that is 50 (low dosage) and 150 (high dosage) times the clinical dosage these results may not indicate a serious allergy concern for humans.

To further evaluate the effects of SBP, we used *Macaca fascicularis* as a model animal because of its close genetic relationship to humans [25]. Using an immunizing dosage that was 50 times the clinical dosage, no abnormalities of toxicological significance were found. Our results showed that SBP could trigger antibody responses as a result of co-immunization with HBV vaccine. Moreover, SBP-adjuvanted vaccine induced an increase in the number of CD4+ T cells, which may correlate with antibody production [26].

The importance of developing safer and better vaccine adjuvants for human use is indisputable, but it is difficult to gain approval for adjuvants as a final product. This study comprehensively evaluated the adjuvanticity and safety of SBP in regard to HBV vaccine. The results demonstrate that SBP could enhance the humoral response elicited by the existing vaccine. Given the encouraging safety data obtained in this study, further evaluation of SBP as a vaccine adjuvant for human use is warranted. This research has the potential to accelerate adjuvant development for HBV vaccine and for other vaccine types in the future.

## Supporting Information

S1 File Tables A-D; Hematology and clinical chemistry parameter changes in rats injected with different agents after 46 days. Values are expressed as mean±standard deviation. * indicates significant increase or decrease in p values.
(DOCX)
