# Supplementary material for: A Pre-Clinical Safety Evaluation of SBP (HBsAg-Binding Protein) Adjuvant for Hepatitis B Vaccine
Source: PLoS One. 2017 Jan 19;12(1):e0170313. doi: 10.1371/journal.pone.0170313 (PMC5245819; doi:10.1371/journal.pone.0170313)
Supplement: S1 File — Values are expressed as mean±standard deviation. * indicates significant increase or decrease in p values. (DOCX) [file pone.0170313.s001.docx]

**A pre-clinical safety evaluation of SBP (HBsAg-binding protein) adjuvant for hepatitis B vaccine**

Jingbo Wang^1^, Caixia Su^1^, Rui Liu^1^, Baoxiu Liu^1^, Inamullah Khan^1^, Jun Xie^1*^, Naishuo Zhu^1*^

^1^Laboratory of Molecular Immunology, State Key Laboratory of Genetic Engineering, Institute of Biomedical Science, School of Life Sciences, Fudan University, Shanghai 200438, China.

*Corresponding author

E-mail: [nzhu@fudan.edu.cn](mailto:nzhu@fudan.edu.cn), xiejun@fudan.edu.cn.

**Table A. Hematology parameter changes in rats injected with different agents after 46 days.**

| **Group** | **Blood cell count** | | | | | | | | | |
| --- | --- | --- | --- | --- | --- | --- | --- | --- | --- | --- |
|  | WBC  (10^9/L)[g] | RBC  (10^12/L)[g] | HGB  (g/L)[g1] | HCT  (%)[g] | MCV  (fL)[g] | MCH  (pg)[g] | MCHC  (g/L)[g] | PLT  (10^9/L)[g] | Neut  (%)[g] | Lymph  (%)[g] |
| **Male rats** |  |  |  |  |  |  |  |  |  |  |
| NS | 8.613±2.645 | 8.893±0.344 | 161.2±6.9 | 49.39±2.02 | 55.55±1.8 | 18.14±0.48 | 326.6 ±7.1 | 1022.9±98.7 | 11.46±3.17 | 83.84±3.46 |
| S (0.5ml) | 8.755±2.451 | 8.893±0.475 | 159.7±1.9 | 49.21±1.90 | 55.39±2.38 | 18.00±0.91 | 324.7±9.3 | 938.3±280.9 | 11.39±4.99 | 84.17±5.08 |
| S (1.5ml) | 10.209±1.134 | 8.863±0.303 | 161.0±5.0 | 49.25±1.84 | 55.56±1.68 | 18.18±0.54 | 327.2±6.6 | 1049.5±182.4 | 9.73±2.91 | 85.56±3.65 |
| H-S (0.5ml) | 9.118±2.246 | 8.921±0.280 | 159.8±5.2 | 48.48±2.15 | 54.34±1.72 | 17.91±0.30 | 329.8±9.1 | 1031.0±124.3 | 12.38±3.58 | 81.27±4.88 |
| H-S (1.5ml) | 9.586±2.117 | 8.717±0.476 | 154.5±5.0 | 47.81±2.30 | 54.94±3.01 | 17.76±0.93 | 323.3±7.6 | 1135.2±114.7 | 10.53±2.27 | 83.58±2.94 |
| **Female rats** |  |  |  |  |  |  |  |  |  |  |
| NS | 5.343±1.978 | 8.279±0.239 | 152.1±3.2 | 45.66±1.42 | 55.23±2.64 | 18.40±0.59 | 333.5± 7.6 | 1196.5±86.9 | 11.22±4.37 | 83.73±4.73 |
| S (0.5ml) | 4.846±1.624 | 8.252±0.377 | 151.2±7.1 | 46.19±2.31 | 56.00±2.31 | 18.32±0.64 | 327.3±7.4 | 1122.5±162.6 | 11.80±3.52 | 82.84±5.06 |
| S (1.5ml) | 7.359±2.264 | 8.230±0.404 | 151.5±7.7 | 45.91±2.44 | 55.79±1.92 | 18.41±0.32 | 330.0±8.1 | 1162.1±79.4 | 10.23±4.08 | 84.77±4.33 |
| H-S (0.5ml) | 6.214±1.721 | 8.178±0.268 | 148.2±3.4 | 44.95±1.32 | 54.95±1.00 | 18.11±0.38 | 329.9±5.8 | 1201.7±165.6 | 14.03±4.20 | 79.23±5.06 |
| H-S (1.5ml) | 7.351±1.455 | 8.141±0.258 | 149.7±6.0 | 45.33±1.84 | 55.68±1.39 | 18.37±0.48 | 330.0±5.8 | 1154.2±119.4 | 11.87±2.84 | 80.90±3.48 |

Values are expressed as mean±standard deviation.

WBC – White Blood Cells, RBC – Red Blood Cells, HGB – Hemoglobin, HCT – Hematocrit, MCV – Mean Corpuscular Volume, MCH – Mean Corpuscular Hemoglobin, MCHC – Mean Corpuscular Hemoglobin Concentration, PLT – Platelets, Neut – [Neutrophile](javascript:void(0);) [granulocyte](javascript:void(0);), Lymph – Lymphocyte.

**Table B.** **Hematology parameter changes in rats injected with different agents after 46 days.**

| **Group** | **Blood cell count** | | | | | | | | | |  |
| --- | --- | --- | --- | --- | --- | --- | --- | --- | --- | --- | --- |
|  | Mono  (%)[g1] | Eos  (%)[g2] | Baso  (%)[g] | Neut  (10^9/L)[g] | Lymph  (10^9/L[g] | Mono  (10^9/L)[g1] | Eos  (10^9/L)[g] | Baso  (10^9/L)[g] | Retic  (10^12/L)[g] | Retic  (%)[g] |  |
| **Male rats** |  |  |  |  |  |  |  |  |  |  |  |
| NS | 1.61±0.55 | 1.18±0.39 | 1.11±0.17 | 0.939±0.279 | 7.256±2.335 | 0.146±0.079 | 0.101±0.048 | 0.097±0.036 | 0.1724±0.0252 | 1.941±0.294 | |
| S (0.5ml) | 1.85±0.77 | 0.75±0.29 | 1.06±0.13 | 0.922±0.225 | 7.448±2.285 | 0.166±0.096 | 0.063±0.031 | 0.093±0.030 | 0.1747±0.0441 | 1.988±0.595 | |
| S (1.5ml) | 1.65±0.23 | 1.08±0.48 | 1.12±0.34 | 0.980±0.267 | 8.751±1.170 | 0.169±0.028 | 0.107±0.037 | 0.113±0.028 | 0.1763±0.0406 | 1.991± 0.466 | |
| H-S (0.5ml) | 1.96±0.47 | 1.45±0.53 | 1.05±0.27 | 1.079±0.271 | 7.447±1.995 | 0.172±0.038 | 0.135±0.057 | 0.097±0.038 | 0.2052±0.0499 | 2.308± 0.596 | |
| H-S (1.5ml) | 2.01±0.53 | 1.53±0.65 | 1.17±0.23 | 1.005±0.318 | 8.011±1.777 | 0.196±0.078 | 0.142±0.057 | 0.116±0.045 | 0.1905±0.0421 | 2.204± 0.546 | |
| **Female rats** |  |  |  |  |  |  |  |  |  |  | |
| NS | 1.51±0.43 | 1.55±0.55 | 1.08±0.14 | 0.583±0.258 | 4.486±1.740 | 0.087±0.049 | 0.080±0.029 | 0.062±0.029 | 0.1668±0.0362 | 2.016±0.447 |  |
| S (0.5ml) | 1.73±1.21 | 1.44±0.74 | 1.10±0.16 | 0.530±0.098 | 4.074±1.609 | 0.074±0.039 | 0.062±0.022 | 0.054±0.018 | 0.1885±0.0314 | 2.285±0.382 |  |
| S (1.5ml) | 1.71±0.44 | 1.21±0.51 | 0.96±0.12 | 0.680±0.117 | 6.314±2.207 | 0.126±0.053 | 0.082±0.027 | 0.071±0.023 | 0.1820±0.0296 | 2.215±0.345 |  |
| H-S (0.5ml) | 2.03±0.71 | 2.43±1.62 | 1.11±0.23 | 0.876*±0.356 | 4.929±1.404 | 0.122±0.039 | 0.145±0.087 | 0.069±0.022 | 0.1978±0.0452 | 2.420±0.571 |  |
| H-S (1.5ml) | 2.33±0.45 | 2.12±0.56 | 1.20±0.36 | 0.875*±0.297 | 5.943±1.150 | 0.170*±0.046 | 0.156*±0.051 | 0.088±0.033 | 0.1780±0.0280 | 2.192±0.373 |  |

Values are expressed as mean±standard deviation. * indicates significant increase or decrease in p values.

Mono – Monocyte, Eos – Eosinophilic granulocyte, Baso – Basophilic granulocyte, Neut – [Neutrophile](javascript:void(0);) [granulocyte](javascript:void(0);), Lymph – Lymphocyte, Retic – Reticulocyte count.

**Table C. Clinical chemistry parameter changes in rats injected with different agents after 46 days.**

| **Group** | **chemistry parameter** | | | | | | | | | |
| --- | --- | --- | --- | --- | --- | --- | --- | --- | --- | --- |
|  | ALT  (U/L) [g] | AST  (U/L) [g] | TP  (g/L)[g] | Alb  (g/L)[g1] | Glb  (g/L)[g] | A/G  [g] | TBil  (µmol/L) [g] | ALP  (U/L) [g] | LDH  (U/L)[g] | CK  (U/L)[g] |
| **Male rats** |  |  |  |  |  |  |  |  |  |  |
| NS | 50.3±14.6 | 142.7±37.4 | 60.37±3.07 | 33.44±1.21 | 26.93±2.19 | 1.24±0.08 | 1.672±0.226 | 204.4±52.7 | 707.0±415.0 | 710.5±303.5 |
| S (0.5ml) | 49.8±15.2 | 143.8±49.7 | 60.64±2.82 | 33.78±1.03 | 26.86±2.33 | 1.27±0.11 | 1.839±0.534 | 215.2±44.9 | 593.3±389.0 | 598.5±273.5 |
| S (1.5ml) | 48.8±10.4 | 140.4±71.5 | 59.38±1.96 | 32.58±0.77 | 26.80±1.38 | 1.22±0.06 | 1.848±0.489 | 185.9±56.9 | 611.5±441.4 | 712.1±597.0 |
| H-S (0.5ml) | 50.9±10.9 | 143.0±38.4 | 59.23±3.67 | 31.84*±0.82 | 27.39±3.02 | 1.18±0.11 | 1.629±0.330 | 180.8±34.1 | 548.6±298.8 | 623.9±290.0 |
| H-S (1.5ml) | 47.5±10.1 | 154.1±30.8 | 59.24±3.36 | 31.11*±1.70 | 28.13±2.08 | 1.11*±0.09 | 1.599± 0.254 | 196.5±45.0 | 802.5±520.4 | 873.5±511.0 |
| **Female rats** |  |  |  |  |  |  |  |  |  |  |
| NS | 34.8±7.3 | 117.4±26.2 | 69.97±4.10 | 38.79±2.15 | 31.18±2.69 | 1.25±0.12 | 1.947±0.306 | 77.0±13.1 | 469.4±279.8 | 549.2±435.7 |
| S (0.5ml) | 37.1±19.6 | 128.9±21.1 | 67.71±2.47 | 38.08±1.51 | 29.63±1.28 | 1.29±0.06 | 2.356±0.493 | 78.8±15.4 | 691.9*±105.4 | 812.6±360.0 |
| S (1.5ml) | 34.7±5.1 | 139.7±27.7 | 66.06±5.03 | 36.43±2.77 | 29.63±2.94 | 1.23±0.12 | 2.195±0.461 | 79.7±20.2 | 832.5±424.6 | 729.8±295.2 |
| H-S (0.5ml) | 38.9±7.8 | 125.6±21.2 | 66.36±4.94 | 36.58±2.18 | 29.78±3.09 | 1.23±0.09 | 2.160±0.475 | 83.4±24.0 | 433.4±268.5 | 409.7±231.3 |
| H-S (1.5ml) | 39.0±16.7 | 143.0±24.8 | 64.76±4.61 | 34.38*±2.24 | 30.38±2.62 | 1.13*±0.05 | 1.716±0.686 | 78.1±22.1 | 635.4±268.7 | 650.1±280.7 |

Values are expressed as mean±standard deviation. * indicates significant increase or decrease in p values.

ALT – Alanine Transaminase, AST – Aspartatetransaminase, TP – Total Protein, Alb – Albumin, Glb – Globulin, A/G – Alb/Glb, TBil – Total Bilirubin, ALP – Alkaline phosphatase, LDH – Lactic Dehydrogenase, CK – creatinekinase.

**Table D. Clinical chemistry parameter changes in rats injected with different agents after 46 days.**

| **Group** | **chemistry parameter** | | | | | | |  | | |
| --- | --- | --- | --- | --- | --- | --- | --- | --- | --- | --- |
|  | Glu  (mmol/L)[g1] | BUN  (mmol/L)[g] | Cre  (µmol/L) [g] | CHO  (mmol/L)[g] | TG  (mmol/L)[g] | Ca  (mmol/L)[g] | P  (mmol/L)[g] | Na+  (mmol/L)[g] | K+  (mmol/L)[g] | Cl-  (mmol/L)[g] |
| **Male** **rats** |  |  |  |  |  |  |  |  |  |  |
| NS | 6.438±0.500 | 5.70±0.79 | 24.3±4.1 | 1.382±0.228 | 0.726±0.314 | 2.557±0.125 | 2.644±0.190 | 141.74±1.77 | 5.128±0.512 | 109.61±2.99 |
| S (0.5ml) | 6.730±0.645 | 6.27±0.86 | 26.3±3.0 | 1.048*±0.186 | 0.737±0.494 | 2.609±0.101 | 2.680±0.213 | 140.56±2.59 | 4.894±0.408 | 108.26±2.73 |
| S (1.5ml) | 6.266±0.737 | 5.98±1.04 | 25.2±2.7 | 1.192±0.313 | 0.660±0.264 | 2.558±0.046 | 2.626±0.140 | 141.88±2.80 | 4.583±0.444 | 111.30±1.85 |
| H-S (0.5ml) | 6.207±0.831 | 6.10±1.11 | 26.5±2.3 | 1.058*±0.247 | 0.605±0.280 | 2.556±0.083 | 2.642±0.142 | 141.15±1.78 | 5.027±0.515 | 110.01±1.81 |
| H-S (1.5ml) | 6.240±0.472 | 6.22±1.07 | 25.9±3.4 | 1.180±0.162 | 0.648±0.276 | 2.545±0.092 | 2.634±0.197 | 141.63±1.84 | 4.774±0.673 | 112.84*±2.43 |
| **Female rats** |  |  |  |  |  |  |  |  |  |  |
| NS | 5.237±0.684 | 6.85±1.08 | 36.5±5.5 | 1.711±0.479 | 0.484±0.353 | 2.622±0.148 | 2.098±0.391 | 142.58±2.08 | 5.103±0.453 | 110.75±1.92 |
| S (0.5ml) | 5.437±0.528 | 6.39±0.69 | 30.5*±4.3 | 1.526±0.555 | 0.365±0.135 | 2.491±0.086 | 1.989±0.267 | 141.89±1.76 | 4.526±0.458 | 112.74±1.03 |
| S (1.5ml) | 5.071±0.662 | 6.64±0.73 | 34.7±3.7 | 1.364±0.454 | 0.411±0.267 | 2.544±0.115 | 2.134±0.293 | 142.31±2.00 | 4.701±0.847 | 113.16*±1.86 |
| H-S (0.5ml) | 5.098±0.575 | 6.49±1.07 | 31.4*±5.3 | 1.524±0.608 | 0.349±0.162 | 2.537±0.150 | 2.174±0.236 | 142.64±1.82 | 5.097±0.538 | 111.34±2.18 |
| H-S (1.5ml) | 5.133±0.667 | 6.90±0.72 | 33.1±3.2 | 1.253±0.370 | 0.341±0.140 | 2.518±0.104 | 2.255±0.291 | 143.09±1.68 | 4.859±0.447 | 114.04*±2.35 |

Values are expressed as mean±standard deviation. * indicates significant increase or decrease in p values.

Cre – creatinine, Glu – glucose, CHO – cholesterol, TG – triglyceride. BUN – Blood [Urea](javascript:void(0);) [Nitrogen](javascript:void(0);), Ca – Calcium, P – PHOS, Na+ - [sodium](javascript:void(0);), K+ - potassium, Cl- - chloride
